# Surfactant-driven optimization of iron-based nanoparticle synthesis: a study on magnetic hyperthermia and endothelial cell uptake†

**DOI:** 10.1039/d3na00540b

**Published:** 2023-10-04

**Authors:** K. Riahi, I. Dirba, Y. Ablets, A. Filatova, S. N. Sultana, E. Adabifiroozjaei, L. Molina-Luna, U. A. Nuber, O. Gutfleisch

**Affiliations:** a Functional Materials, Institute of Materials Science, Technical University of Darmstadt Peter-Grünberg-Str. 16 64287 Darmstadt Germany kalthoum.riahi@tu-darmstadt.de; b Advanced Electron Microscopy Division, Institute of Materials Science, Technical University of Darmstadt Peter-Grünberg-Str. 22 64287 Darmstadt Germany; c Stem Cell and Developmental Biology, Technical University of Darmstadt 64287 Darmstadt Germany

## Abstract

This work examines the effect of changing the ratio of different surfactants in single-core iron-based nanoparticles with respect to their specific absorption rate in the context of magnetic hyperthermia and cellular uptake by human umbilical vein endothelial cells (HUVEC). Three types of magnetic nanoparticles were synthesized by separately adding oleic acid or oleylamine or a mixture of both (oleic acid/oleylamine) as surfactants. A carefully controlled thermal decomposition synthesis process led to monodispersed nanoparticles with a narrow size distribution. Spherical-shaped nanoparticles were mainly obtained for those synthesized with oleic acid, while the shape changed upon adding oleylamine. The combined use of oleic acid and oleylamine as surfactants in single-core iron-based nanoparticles resulted in a substantial saturation magnetization, reaching up to 140 A m^2^ kg^−1^ at room temperature. The interplay between these surfactants played a crucial role in achieving this high magnetic saturation. By modifying the surface of the magnetic nanoparticles using a mixture of two surfactants, the magnetic fluid hyperthermia heating rate was significantly improved compared to using a single surfactant type. This improvement can be attributed to the larger effective anisotropy achieved through the modification with both (oleic acid/oleylamine). The mixture of surfactants enhances the control of interparticle distance and influences the strength of dipolar interactions, ultimately leading to enhanced heating efficiency. Functionalization of the oleic acid-coated nanoparticles with trimethoxysilane results in the formation of a core–shell structure Fe@Fe_3_O_4_, showing exchange bias (EB) associated with the exchange anisotropy between the shell and the core. The biomedical relevance of our synthesized Fe@Fe_3_O_4_ nanoparticles was demonstrated by their efficient uptake by human umbilical vein endothelial cells (HUVECs) in a concentration-dependent manner. This remarkable cellular uptake highlights the potential of these nanoparticles in biomedical applications.

## Abstract 2

This work examines the effect of changing the ratio of different surfactants in single-core iron-based nanoparticles in the context of magnetic hyperthermia and cellular uptake by human umbilical vein endothelial cells.

## Graphical abstract

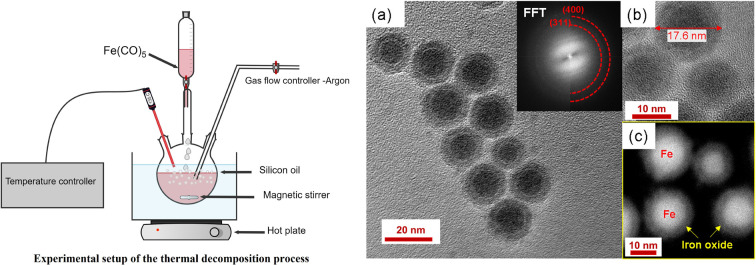

## 1 Introduction

When colloidal magnetic nanoparticles (MNPs) are exposed to an alternating magnetic field (AMF), they can convert electromagnetic energy into heat. This technique is known as magnetic fluid hyperthermia (MFH), and it involves the application of a low electromagnetic field to locally heat a targeted volume. MFH is a versatile method that has already been used in various medical applications, including the treatment of malignant tumors and as a way to trigger controlled drug release.^1–3^ This is achieved by exploiting the unique properties of MNPs coated with a polymer, where MNPs act as heat generators in response to alternating magnetic field stimuli, while the polymer serves as a mechanism for drug storage and release, as well as providing stability and biocompatibility.^4^ In addition, MFH is being studied as a warming technique for frozen organs in biobanking, where organs can be uniformly perfused, vitrified, and rapidly rewarmed to room temperature.^5^

Magnetite (Fe_3_O_4_) and maghemite (γ-Fe_2_O_3_) nanoparticles are among the most studied MNPs for potential magnetic heating applications due to their tunable magnetic properties, intrinsic biocompatibility, and stable suspension in the superparamagnetic regime. However, the low heating efficiency of commercially available single-core iron oxide nanoparticles with specific absorption rate (SAR)^6^ values of a few hundred watts per gram poses a major challenge for clinical applications. This low SAR is attributed to factors such as small particle sizes,^7^ agglomeration of nanoparticles due to inappropriate surface coating, and the surface spin disorder of nanoparticles, which can cause a magnetically inactive surface layer.^8^ Additionally, the reduction in the saturation magnetization of iron oxide nanoparticles with size also leads to a decrease in heating efficiency. To address these issues, the interface between the core and surface of MNPs can be controlled using different surfactants. By binding to the surface of the nanoparticles, surfactants can reduce surface disorder, change surface anisotropy, and affect magnetization. The thickness and chemical nature of the organic layer around MNPs also contribute to controlling the distance between particles and thus the strength of dipolar interactions, which affect the effective anisotropy of the nanoparticle assembly.^9^ Therefore, the magnetic properties of MNPs can be tuned by selecting appropriate surfactants with specific functional groups and chain lengths.^10^

Several chemical techniques can be used to adjust the size, shape, and magnetic-phase properties of MNPs, and to prevent their agglomeration, with varying types and concentrations of surfactants being a key component.^11^ In fact, MNPs are usually stabilized by organic surfactants adsorbed on their surfaces, providing steric repulsion and preventing neighboring MNPs from approaching each other.^12^ Oleic acid (OA) (C_18_H_34_O_2_) and oleylamine (OY) (C_18_H_37_N) are commonly used surfactants in chemical synthesis to produce fine nanoparticles. OA is preferred as an organic surfactant because it can strongly bind to oxide surfaces as a protective monolayer while allowing easy functionalization by ligand exchange. This results in the synthesis of monodisperse and highly uniform nanoparticles.^13^ OY, on the other hand, is a long-chain primary alkylamine that acts as an electron donor at elevated temperatures. It is liquid at room temperature, which simplifies washing procedures after the chemical synthesis of MNPs.^14^ It is not only a surfactant, but can also act as a solvent and mild reducing agent.^15^ The combination of oleic acid and oleylamine is a common surfactant used to protect MNPs from van der Waals attraction, minimize interparticle interactions, and limit growth by acting as a steric barrier to mass transfer. This combination is frequently chosen for the shape-controlled synthesis of colloidal inorganic nanocrystals.^16,17^

In this study, we propose to vary the ratio of surfactant used to synthesize iron-based nanoparticles, testing three different ratios; a mixture of oleic acid and oleylamine (1 : 1), and separately oleic acid (1 : 0), oleylamine (0 : 1), and assess their magnetic properties and MFH heating ability. In fact, pure iron is known for its high saturation magnetization (220 emu g^−1^) compared to other magnetic materials.^18^ This should lead to enhanced heating power in MFH.^18^ However, it is difficult to prepare pure Fe nanoparticles due to oxidation, leading to various iron oxide nanoparticles. In this context, surfactants have a dual role – as capping agents for nanoparticles by binding their functional groups to the surface, and as dispersing liquids for the nanoparticles. The combined mixture of oleic acid and oleylamine can create an acid–base complex and hydrogen bonding to modify the composition, viscosity, and functionality of the mixtures for stabilizing magnetic nanoparticles.^19^ To achieve aqueous dispersions the MNPs were functionalized through a ligand exchange reaction with “trimethoxysilane” and transferred to water for cellular uptake experiments.

In this study, human umbilical vein endothelial cells (HUVEC)^20^ were used to elucidate the internalization of functionalized magnetic nanoparticles by cells, highlighting their potential for biomedical applications. In the case of a systemic application of iron-based nanoparticles for MFH-based treatment of malignant tumors, *i.e.* an injection into the blood circulation, the particles will be in immediate contact with endothelial cells, which form the inner cell layer of blood vessels. While numerous studies have thoroughly examined the cellular uptake of magnetic nanoparticles by cancer cells,^21–23^ our findings provide valuable insights into the internalization of these nanoparticles by endothelial cells. Tumor endothelial cells differ from endothelial cells in normal tissues, including the presentation of certain surface molecules.^24,25^ Targeting tumor endothelial cells will require further modifications of the MNP surface, *e.g.*, aptamer functionalization, to achieve a specific binding to tumor endothelial markers.

## 2 Materials and methods

### 2.1 Synthesis of magnetic nanoparticles

**Fig. 1:**
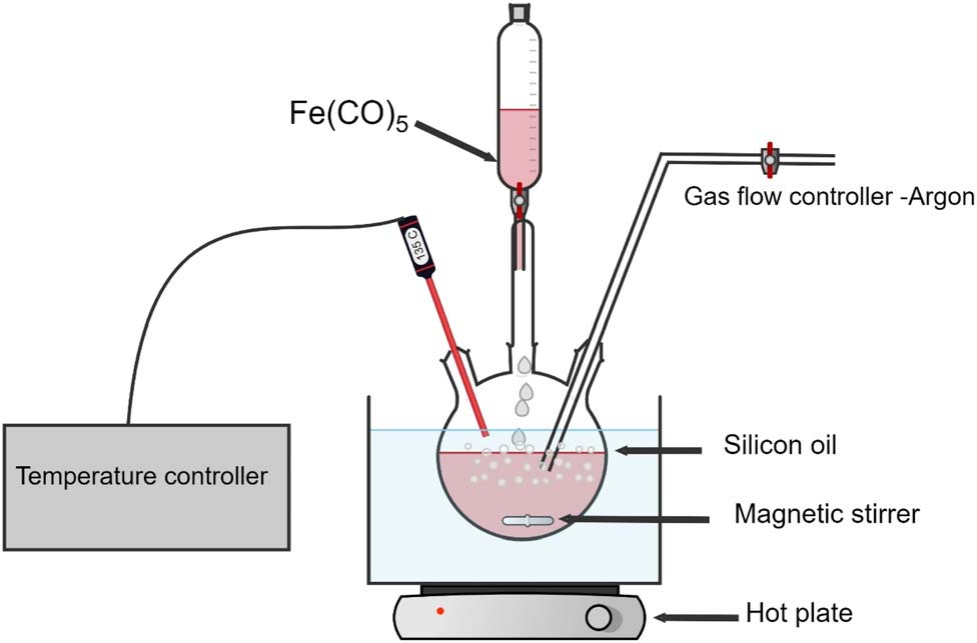
Experimental setup of the thermal decomposition process.

Monodispersed iron-based nanoparticles were synthesized *via* thermal decomposition of Fe(CO)_5_ (10 ml, >99.99% Sigma-Aldrich) in a three-neck round-bottom flask filled with kerosene (Sigma-Aldrich, reagent grade) as a solvent. The flask was heated in an oil bath (crystallizer filled with silicon oil). Precise temperature control was realized by a thermocouple in the flask with a tip immersed directly in the liquid phase. Before each experiment, the apparatus was tested for vacuum and pre-heated with a heat gun with continuous pumping to avoid contamination. An example of the experimental setup is shown in oleic acid (OA) (pure, 100%, Bernd Kraft) and oleylamine (OY) (70%, Sigma-Aldrich) were added as surfactants to stabilize the nanoparticles. Three distinct samples were prepared by altering the oleic acid and oleylamine surfactant ratios (OA : OY) to 1 : 0, 0 : 1, and 1 : 1 (further in the text referred to as NPs-OA, NPs-OY and NPs-OA/OY respectively), and each surfactant ratio was added to 50 ml of kerosene as a carrier liquid. The carrier liquid and surfactant mixture was continuously purged with Ar gas while the main precursor, iron pentacarbonyl Fe(CO)_5_, was added dropwise. After synthesis, nanoparticles were stored in an Ar-filled glovebox to avoid oxidation (Fig. 1).

### 2.2 Post-synthesis processing: washing and drying

The obtained MNPs were washed with isopropanol (spec. laboratory reagent, >95%, Fisher Scientific) followed by magnetic separation. The process was repeated in an Ar-filled glovebox until isopropanol in suspension became transparent and did not contain any visible traces of the organic residues. The next essential step was drying of the MNPs to remove residual isopropanol. This procedure was performed by continuous pumping at room temperature for 2 hours. A cold trap, filled with liquid nitrogen was used to protect the pump. Finally, the obtained MNPs were dispersed in hexane for storage and for heating rate measurements.

### 2.3 Surface functionalization by ligand-exchange of magnetic nanoparticles-oleic acid (1 : 0) with silane

MNPs synthesized with oleic acid as a surfactant were subsequently functionalized as follows. The surface functionalization was achieved by the protocol described by Bolemen *et al.*^26^ MNPs in heptane (Sigma-Aldrich, anhydrous, 99%) solution (100 mg ml^−1^) were mixed with toluene (Sigma-Aldrich, anhydrous, 99.8%), triethylamine (Sigma-Aldrich, ≥99%), MilliQ water, and 2-[methoxy(polyethyleneoxy)propyl]trimethoxysilane. The functionalized particles were labeled as silane-coated nanoparticles. The mixture was kept in an ultrasonic bath for 5 h. Thereafter, the silane-coated nanoparticles were precipitated using an Nd–Fe–B magnet, and the supernatant was decanted. The sample was dried under vacuum and dissolved in MilliQ water for further experiments.

### 2.4 Nanoparticle uptake by human umbilical vein endothelial cells

#### 2.4.1 Culturing and magnetic labeling of HUVECs

Human umbilical vein endothelial (HUVEC) cells (cAP-0001GFP, Angio-Proteomie) were cultured in Endothelial Cell Medium (ECM, P60104, Innoprot). For the uptake experiments, the cells were seeded into a 6-well plate at a density of 0.15 × 106 cells per well. The next day, the medium was exchanged with ECM containing 10, 50, 100, 200, and 400 μg ml^−1^ silane-coated nanoparticles. The medium for control cells was exchanged with ECM without additives.

#### 2.4.2 Prussian blue staining

24 h after magnetic labeling, the cells were collected by trypsinization, washed once with phosphate-buffered saline (PBS), and plated onto poly-lysine-coated glass slides using a cytospin (4 min, 1000 rpm). After drying the slides, the cells on the slides were fixed with ice-cold 4% paraformaldehyde for 30 minutes followed by two washing steps in PBS for 10 min each and incubation with a staining solution for 20 min. The staining solution was prepared immediately before use by mixing equal volumes of 20% hydrochloric acid and 10% potassium ferrocyanide solution. The slides were washed three times in distilled water and counterstained with nuclear fast red (Merck #15939) for 5 minutes. Thereafter, the slides were rinsed twice in distilled water, dehydrated in 95% ethanol and 2 changes of 100% ethanol, and mounted with Kaiser's Glycerine-Gelatine (#1.09242.0100, Merck). Images were acquired using a Nikon SMZ 1500 microscope.

## 3 Characterization of magnetic nanoparticles

### 3.1 Particle size and crystal structure

A few microliters of nanoparticles were drop-cast onto carbon-coated copper grids, and dried at room temperature for 12 h, after being ultra-sonicated for 10 min. The core size and morphology of nanoparticles were investigated with an FEI CM20 ST transmission electron microscope (TEM) operated at an acceleration voltage of 200 kV as well as a 200 kV Jeol JEM 2100F STEM. The image processing and size analysis distribution were determined using the “ImageJ” software. The identification of the selected area (electron) diffraction (SAED) images was processed by CrysTBox ringGUI.

### 3.2 Magnetic characterization

The DC magnetization of each prepared sample (dried powder) as a function of the magnetic field at room temperature was measured under 2 T using a vibrating sample magnetometer (VSM) (7400 Series, Lake Shore). ZFC/FC magnetization curves from room temperature to 5 K were performed using a MPMS3 SQUID magnetometer (Quantum Design). The same device was used for exchange bias measurements, in which the sample was cooled down to 5 K under different applied fields and then the *M*(*H*) was measured.

### 3.3 Magnetothermal measurements

To characterize the heating of magnetic nanoparticles under an AC magnetic field, the heating ability was determined from temperature–time profiles and computed as heat generation per mass of NPs in W g^−1^. The magnetic heating properties of colloid samples were measured using an AC magnetic field applicator (DM100 System, developed by Nanoscale Biomagnetics, Spain) with a vacuum-insulated calorimeter and a water cooling system. 1 ml of nanoparticles with a concentration of 5 mg ml^−1^ suspension in hexane (pure, >95%, Kraft) incorporated with a fiber-optic temperature sensor located in the middle of the glass suspension bottle, was used for the heating rate measurements. The data was processed using the MANIAC v1.0 software.

## 4 Results and discussion

### 4.1 Characterization of the initial magnetic nanoparticles without functionalization

**Fig. 2:**
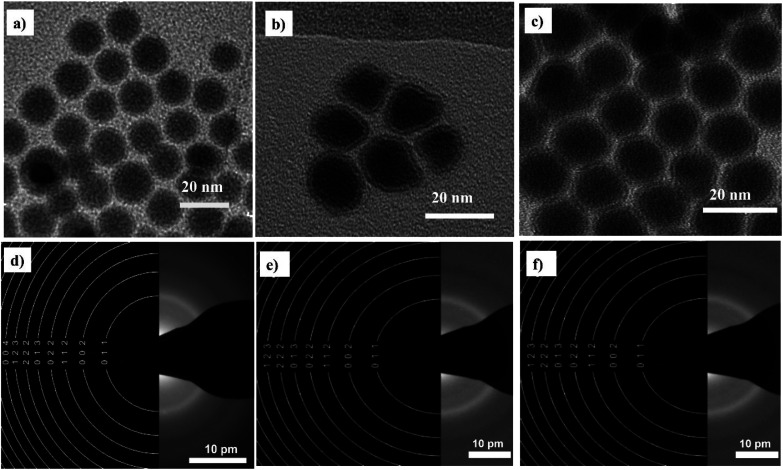
Bright field TEM images of the nanoparticles before functionalization and selected-area electron diffraction (SAED) pattern (a and d) for NP-OA; (b and e) for NP-OY and (c and f) for NP-OA/OY.

Fig. 2 shows that the synthesized nanoparticles are monodisperse, single core, and exhibit a narrow size distribution that is well-fitted by a log-normal distribution function. The NPs-OA are spherical with a 13.45 ± 1.2 nm particle size. However, the nanoparticles prepared only with oleylamine show an irregular shape with a mean size of 14.65 ± 1.54 nm. The NPs-OA/OY with the mixture of both surfactants is quasi-spherical with a mean size of 15.7 ± 1.64 nm. These TEM results confirm that the surfactants oleic acid and oleylamine were efficient to avoid nanoparticle aggregation, and the distance between neighboring nanoparticles is uniform. A passivating oxidation layer (the brighter layer visible in high magnification images) can be distinguished from the core and most likely forms after reaction with the surfactant.^27,28^ The thickness of the oxidation layer is around 4.9 ± 0.5 nm, 2.5 ± 0.2 nm, and 1.6 ± 0.17 nm for NPs-OA, NPs-OY, and NPs-OA/OY, respectively. Diffraction ring patterns match with the α-Fe phase in the three samples (see Fig. 2).

**Fig. 3:**
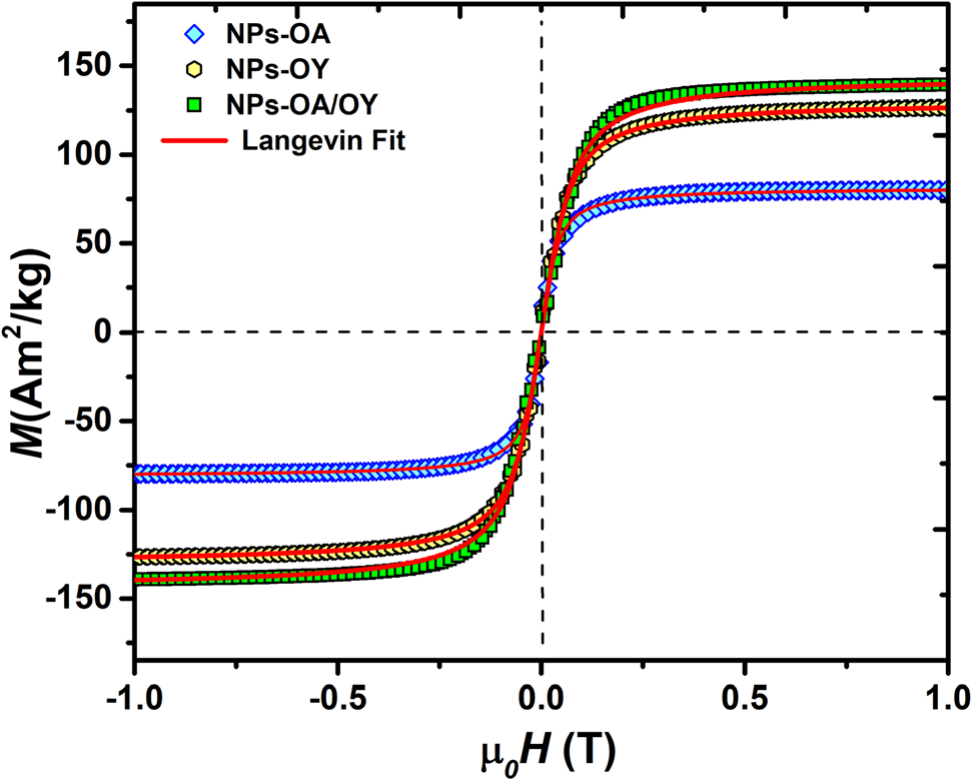
Room temperature magnetization of the Fe-based NPs with different surfactants.

The room temperature *M*–*H* loops (Fig. 3) exhibit very small and negligible values of coercivity and remanence, which reveals the nearly superparamagnetic behavior of the prepared nanoparticles. This observation is further verified by fitting the magnetization curve with the Langevin function:^29^
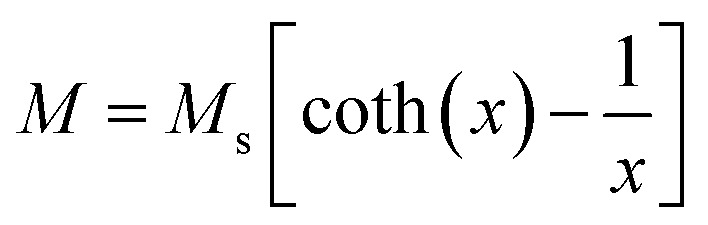
where *M*_s_ is the saturation magnetization, and *x* = *μ*_0_*mH*/*k*_B_*T*, where *μ*_0_ is the vacuum permeability, *k*_B_ is the Boltzmann constant, *m* is the magnetic moment of the nanoparticles and *T* is the absolute temperature.

The obtained *M*_s_ values are 80, 127, and 140 A m^2^ kg^−1^ for NPs-OA, NPs-OY, and NPs-OA/OY, respectively. These values are less than the values reported in the literature by Dirba *et al.* for Fe nanoparticles produced by hydrogen reduction of γ-Fe_2_O_3_.^30^ However, they are high compared to the conventional iron oxides. The low *M*_s_ for the NPs-OA compared to the other MNPs is due to the presence of a thick passivating oxidation layer, as evident from the TEM images. It confirms that oleic acid has a much stronger covalent bond with iron nanoparticles due to high oxophilicity (the tendency to form oxides) which leads to the formation of iron/iron-oxides core/shell nanoparticles. In contrast, oleylamine binds weakly to the Fe nanoparticle's surface^31^ in a single motif.^32^ However oleic acid has different possible binding motifs “monodentrate, bridged, or chelating”. Considering the three binding modes it is clear that oleic acid has a greater probability of binding to nanoparticles' surface than oleylamine.^32^

**Fig. 4:**
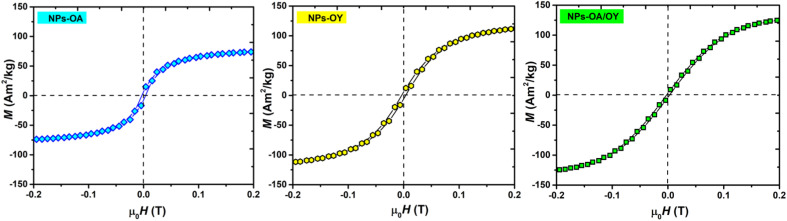
Zoom-in *M*(*H*) curves for the nanoparticles with different surfactants.

Hysteresis loops measured at low applied fields (Fig. 4) show very small coercivity and remanence, possibly due to the interactions between the particles since dried powders were used in the VSM measurements.^27^

**Fig. 5:**
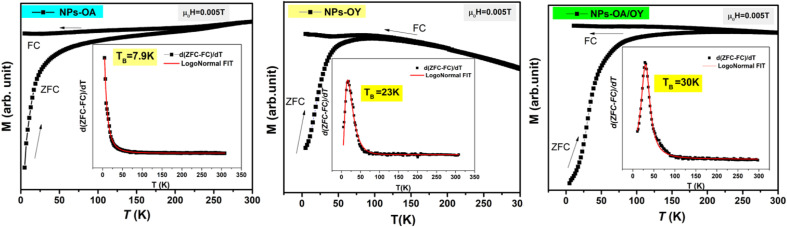
ZFC/FC magnetization curves and the blocking temperature *T*_B_.

The ZFC/FC magnetization curves of the synthesized nanoparticles are shown in Fig. 5. As expected, the blocking temperature (*T*_B_) is increasing with nanoparticle size (Table 1) consistent with the data reported elsewhere.^33^ The average value of *T*_B_ was determined by the method based on the *T* derivative of the difference between ZFC and FC curves with respect to temperature d[*M*_ZFC_ − *M*_FC_]/d*T* proposed by Micha *et al.*^34^ and confirmed later to be the best determination's method of *T*_B_ by Bruvera *et al.*^35^ The effective anisotropy constant *K*_eff_ is an important parameter affecting the heating performance of MNPs. *K*_eff_ is calculated using *T*_B_ = *K*_eff_*V*/25 *k*_B_, where *V* is the particle volume.^36^ In their work, Vasilakaki *et al.*^37^ pointed out that the acidity of the coordination ligand was found to be the major factor affecting the reduction of the anisotropy. Their statement is consistent with the lowest value of *K*_eff_ of NPs-OA in this work (Table 1). When Fe cations at the surface are coordinated to oleic acid, the crystal field splitting energy is high, and the spin–orbit coupling that increases anisotropy becomes smaller. Moreover, the coordinated surfactant increases the interparticle distance, and both dipolar and exchange interactions between neighboring particles are minimized.

**Table 1:** Blocking temperature *T*_B_, size of nanoparticles, the thickness of oxidation layer, the magnetization saturation *M*_s_ and the effective anisotropy *K*_eff_

	*T*_B_ (K)	NP size TEM (nm)	Oxidation layer (nm)	*M*_s_ (A m^2^ kg^−1^)	*K*_eff_ (kJ m^−3^)
NPs-OA	7.8	13.45 ± 1.2	4.9	80	2.11
NPs-OY	23	14.65 ± 1.54	2.5	127	4.82
NPs-OA/OY	30	15.7 ± 1.64	1.6	140	5.11

In order to determine the heating efficiency of these nanoparticles, we have carried out calorimetric magnetic fluid hyperthermia heating rate measurements at AC magnetic field 30 mT, with different frequencies (98 kHz, 200 kHz, and 400 kHz). All measurements were within the safety limits as suggested by Hergt and Dutz criterion of *H* × *f* ≤ 5 × 109 A ms^−1^ for clinical application of MFH.^38^

**Fig. 6:**
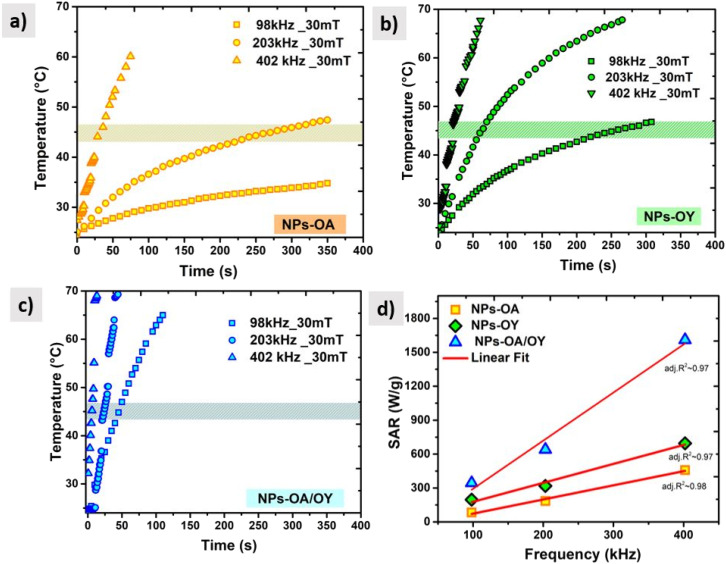
AC heating curves (a) NPs-OA (b) NPs-OY (c) NPs-OA-OY, and (d) the specific absorption rate SAR as a function of frequency.

**Fig. 7:**
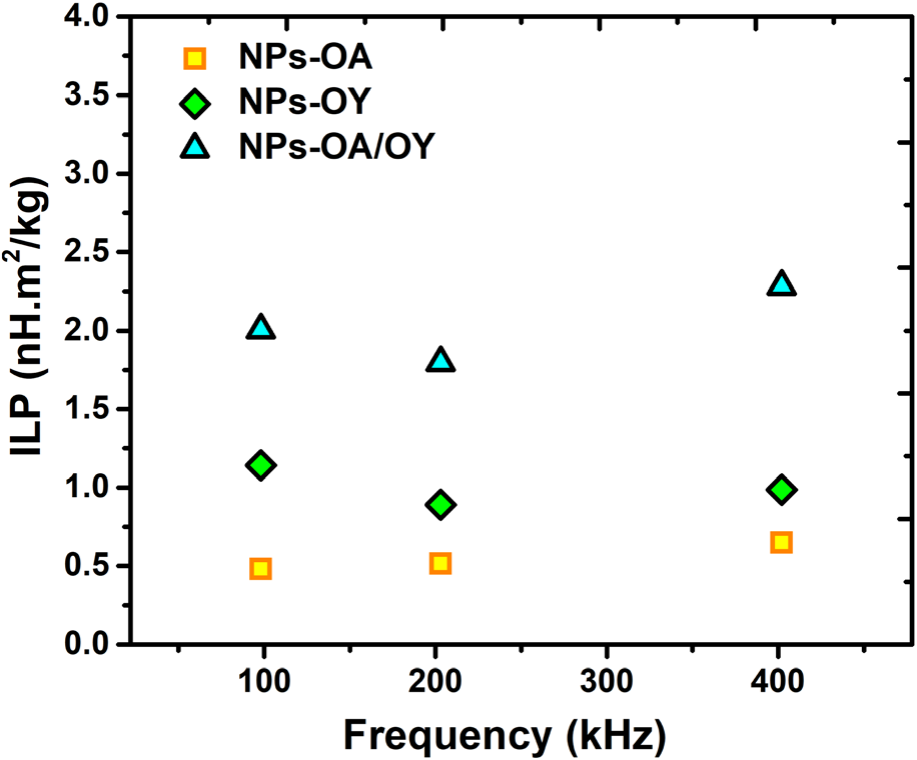
The intrinsic loss power (ILP) values of different MNPs under different frequencies.

For reliable extraction of SAR, the heating curve is fitted using the phenomenological Box–Lucas method with the following expression:^39^ Δ*T* = *a*(1 − e^−*b*(*t*−*t*_0_)^) with *a* and *b* as the fitting parameters. *a* is the initial slope of the heating curve, and *b* is a constant describing the cooling rate. SAR values are then calculated as SAR = *abC*/*m*_MNPs_, where *C* is the specific heat capacity of the solution (hexane), and *m*_MNPs_ is the mass of MNPs. The intrinsic loss power parameter (ILP, nH m^2^ kg^−1^) enables normalization of SAR values measured at different magnetic field amplitudes/frequencies. Fig. 6 illustrates the heating curves for the different nanoparticles, which display a steady increase in temperature over time. At a fixed AC field amplitude, the heating curves exhibit an upward trend as the frequency rises from 98 to 402 kHz. Notably, at all frequencies, the NPs-OA show the slowest and the NPs-OA/OY have the highest heating rates. This results in similar trends in the calculated SAR and ILP values as shown in Fig. 6d and 7 respectively. The enhancement in heating efficiency from changing the ratio of surfactants correlates with *M*_s_ of the nanoparticles. However, since the energy dissipation in nanoparticles is a complex process that depends on various factors such as anisotropy and viscosity of the medium, no clear conclusion can be drawn.

### 4.2 Characterization of NPs-oleic acid after surface functionalization

**Fig. 8:**
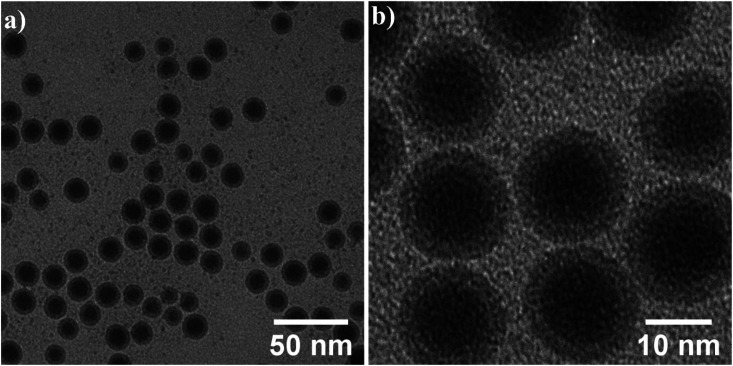
(a) Low and (b) high magnification bright field TEM images of the nanoparticles after surface functionalization.

In the previous section, magnetic nanoparticles were dispersed in hexane, since due to the presence of organic ligands oleic acid and oleylamine on the NPs surface they are hydrophobic. For colloidal stability and experiments with cells, high-quality water-dispersible nanoparticles are essential. This goal was achieved by the introduction of hydrophilic functional groups onto the nanoparticles' surface. The ligand exchange surface functionalization with silane has been performed as described above in section (2.3). TEM images after the ligand exchange are shown in Fig. 8. The core–shell structure of silane-coated nanoparticles and the interface between the Fe core and iron oxides shell can be seen and the shell thickness has roughly doubled in comparison to the initial NPs.

**Fig. 9:**
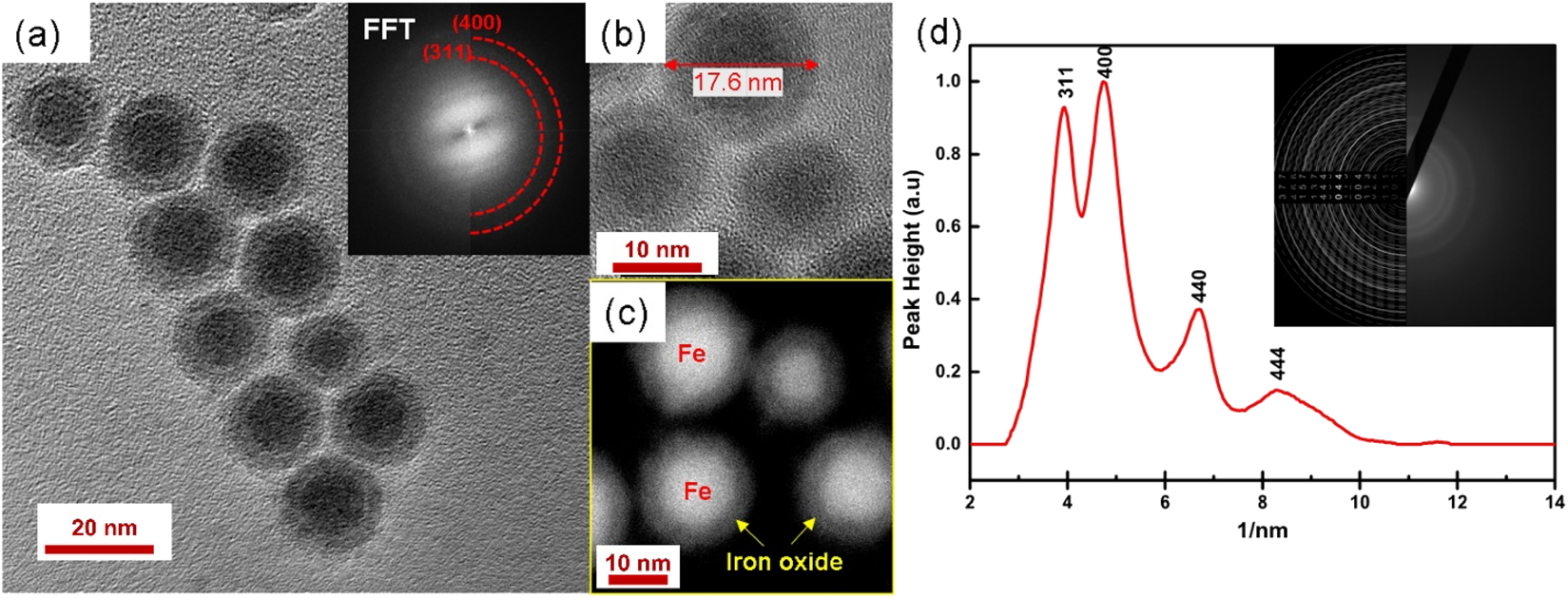
(a) HRTEM image of nanoparticles dispersed in water after surface functionalization. The inset shows the corresponding FFT; high magnification image (b) and STEM image (c) show the core/shell structure in more detail; 2D radial plot (d) of SAED pattern (inset) identifies the shell as magnetite Fe_3_O_4_.

More detailed analysis is shown in Fig. 9. High magnification image (b) and STEM image (c) show the core/shell structure in more detail, the inset shows the corresponding FFT. 2D radial plot (d) of the SAED pattern (inset) identifies the shell as magnetite Fe_3_O_4_. However, distinguishing between magnetite and hematite is not straightforward. To summarize, spherical, water-dispersed core/shell Fe/Fe_3_O_4_ nanoparticles with narrow size distribution have been successfully prepared.

**Fig. 10:**
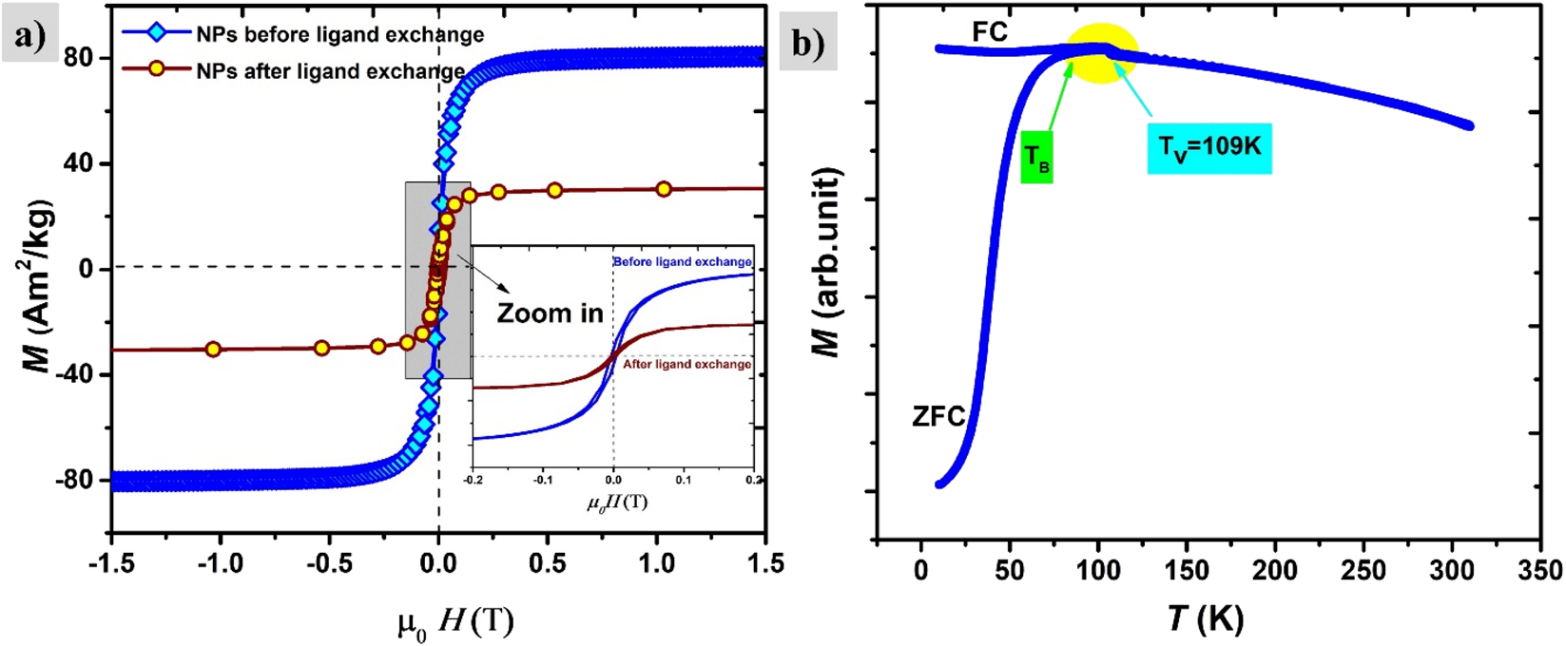
(a) Magnetization curves of nanoparticles before and after ligand exchange, zoom in (the inset). (b) The ZFZ/FC curve of nanoparticles after ligand exchange, showing the blocking temperature *T*_B_ and *T*_V_ Verwey transition.

The saturation magnetization before surface functionalization in the applied magnetic field of μ_0_*H* = 1 T is 80 A m^2^ kg^−1^ which is much lower than bulk iron due to the passivation layer as well as the nonmagnetic surfactant contribution. However, after functionalization with silane, the magnetic saturation dramatically diminishes to 30 A m^2^ kg^−1^ as shown in Fig. 10. The reported magnetization values are presented in terms of the total mass of the sample without subtraction of the mass of the organic shell. The decrease in saturation magnetization is attributed to the shell contribution. The appearance of the Verwey transition *T*_V_ = 109 K (` ^40^) confirms the presence of the magnetite phase as observed in TEM images. In addition, the surface spins of nanoparticles are more susceptible to canting than those inside the cores.^41^ They are coupled with core spins *via* exchange interactions, surface spin orientations occur in specific angles with respect to core spins which are called canting angles. The canted spins have multiple configurations for each orientation of the core magnetization which, might be a reason also for decreasing magnetization after the ligand exchange.^42^

**Fig. 11:**
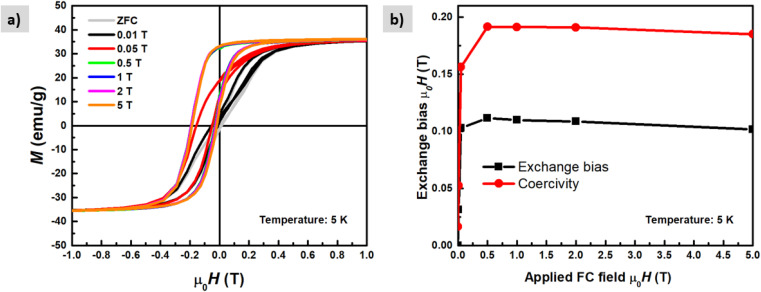
*M*(*H*) loops (a) measured at 5 K temperature after field cooling in different applied magnetic fields up to μ_0_*H* = 5 T. (b) Exchange bias field and coercivity as a function of the applied magnetic field during cooling.

The magnetic properties of the shell identified previously as magnetite Fe_3_O_4_ have short-range super-exchange interactions which give rise to antiferromagnetic alignment.^43^*M*–*H* curves were also measured under different cooling fields at low temperatures (5 K) in order to determine the existence of exchange coupling at the core/shell interface. In Fig. 11a pronounced shift of the hysteresis loops towards negative fields can be observed due to the exchange bias effect. Both exchange bias *μ*_0_*H*_E_ and coercivity *μ*_0_*H*_C_ are continuously increasing with the applied magnetic field and reach maximum at 0.5 T. The obtained results are consistent with those from Kaur *et al.*, who explained that the frozen interfacial spins can relax into random orientations after multiple cycles of hysteresis with subsequent loss of *H*_E_.^44^ We observe a change in the magnetization at low fields which is attributed to the unidirectional alignment of frozen interfacial spins during the FC, which provides maximum exchange coupling between the core and the shell.^45^ The origin of exchange coupling is still not well explained for core–shell magnetic nanoparticles. For instance, Leighton *et al.*,^46^ attributed it to defects present in the Fe core. However, Kaur *et al.*, confirmed that the exchange bias effect purely arises from coupling at the core/shell interface during the field cooling process, which has been explained in terms of the existence of disordered surface spins that freeze in a spin glasslike state.^44^

### 4.3 Cellular uptake study of NPs-oleic acid after surface functionalization

**Fig. 12:**
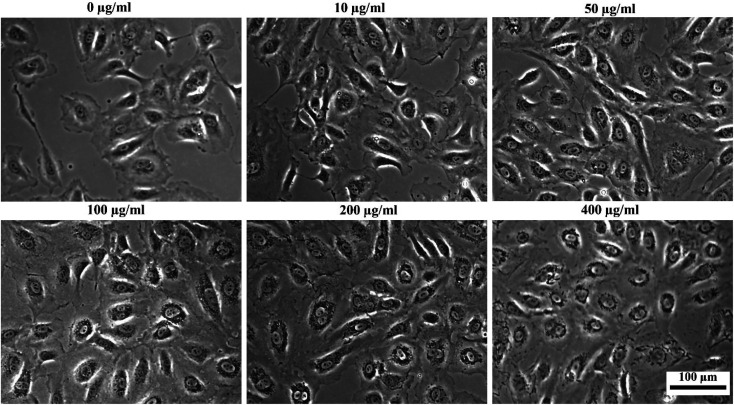
Bright-field optical microscopy images of HUVECs after incubation with different concentrations of silane-coated nanoparticles overnight (note the tiny dark particles inside cells).

**Fig. 13:**
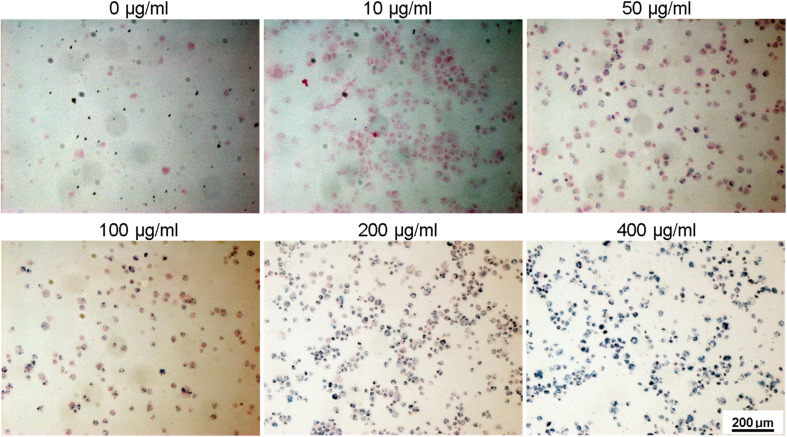
Ferric iron staining (Prussian Blue) was performed with HUVECs incubated with various concentrations of silane coated MNPs ranging from 10 to 400 μg ml^−1^ for 24 h. Dark blue color indicates the stained nanoparticles within the cells.

To assess the suitability of the produced nanoparticles for biomedical applications, HUVECs were incubated with various concentrations (0, 10, 50, 100, 200, and 400 μg ml^−1^) of silane-coated nanoparticles for 24 hours. Nanoparticles were taken up by HUVECs in a concentration-dependent manner and no free nanoparticles were observed in the medium after 24 h incubation. The nanoparticles could be retained inside the cells for at least two weeks. Fig. 12 shows bright field optical microscopy images indicating that the number of internalized nanoparticles in HUVECs is positively correlated with the concentration of nanoparticles added to the cell culture. These results were further confirmed by Prussian Blue staining which is used for the detection of ferric iron in tissues and cells (Fig. 13). With increasing concentrations of nanoparticles incubated with HUVECs for 24 h, the number of labeled cells as well as the amount of the incorporated silane nanoparticles increased. At a concentration of 400 μg ml^−1^, the cells were observed to be densely packed with aggregated nanoparticles. In addition, we have also captured a video (ESI†) demonstrating that after uptake of the nanoparticles, cells can be easily guided by an applied external magnetic field.

## 5 Conclusions

In conclusion, our study has yielded significant findings in the synthesis and characterization of monodisperse iron-based nanoparticles with a narrow size distribution. We systematically investigated the influence of different surfactants, specifically oleic acid and oleylamine, on the particle size, shape, magnetic properties, and magnetic fluid hyperthermia (MFH) heating ability. The saturation magnetization values obtained for these nanoparticles were found to be notably higher compared to conventional iron oxides. We observed a reduction in magnetization for nanoparticles synthesized with oleic acid as the surfactant, which can be attributed to the formation of an oxide layer as evident from TEM investigations. In contrast, nanoparticles coated with oleylamine or a mixture of both exhibited higher saturation magnetization values. These results indicate that when maximizing magnetization and MFH heating power is a priority, the use of pure oleic acid as a surfactant should be avoided. Our study has revealed that the SAR values measured by calorimetry correlate with *M*_s_ of the nanoparticles. Since the energy dissipation in nanoparticles is a complex process that depends on various parameters, additional characterization such as AC magnetometry is necessary to unambiguously explain these results. In order to make the iron nanoparticles water-dispersible and suitable for experiments with cells, hydrophilic functional groups were introduced onto the surface through ligand exchange with covalently bonded ligands trimethoxysilane. This exchange resulted in the formation of spherical core–shell Fe/Fe_3_O_4_ nanoparticles with a doubled shell thickness leading to a reduction in magnetization. Additionally, a pronounced exchange bias effect was observed in the *M*–*H* curves at low temperatures due to exchange coupling between the core and the shell. Importantly, we successfully demonstrated the biomedical applicability of the functionalized silane-coated particles through their concentration-dependent uptake by HUVECs.

In summary, the choice of surfactant plays a critical role in determining the magnetic heating of the nanoparticles, and it should be carefully optimized for specific biomedical applications.

## 6 Future work

We will extend this study using a different method to analyse magnetic hyperthermia, specifically by directly measuring dynamic hysteresis loops using AC magnetometry. This approach will provide unique insights into the dynamic behavior of magnetic nanoparticles which are not achievable through calorimetric measurements. Additionally, we aim to transfer the NPs-OA/OY, which contain a mixture of both surfactants and exhibit high saturation magnetization, into a water-based solution. We will carefully optimize the thickness of the coating to maintain both the high saturation magnetization and specific absorption rate (SAR). In a subsequent phase of our research, we will test these particles with various cancer cell lines to assess their cellular uptake and compare them with the results obtained in the current study.

## Author contributions

K. Riahi: writing – original draft preparation, data processing, analysis, review & editing. I. Dirba, Y. Ablets: synthesis and preparation of the materials, review & editing. K. Riahi, I. Dirba and Y. Ablets: testing, characterization of the materials, review & editing. S. N. Sultana, E. Adabifiroozj, and L. Molina-Luna: imaging of materials with a high-resolution transmission electron microscopy, review & editing. A. Filatova: cell biology experiments, review & editing. U. A. Nuber: advice, review & editing. O. Gutfleisch: supervision, review & and editing, project administration, funding acquisition.

## Conflicts of interest

All authors declare no conflict of interest.

## Supplementary Material

NA-005-D3NA00540B-s001
